# Dynamics of high frequency brain activity

**DOI:** 10.1038/s41598-017-15966-6

**Published:** 2017-11-17

**Authors:** Steven X. Moffett, Sean M. O’Malley, Shushuang Man, Dawei Hong, Joseph V. Martin

**Affiliations:** 1 Center for Computational and Integrative Biology, Rutgers University, Camden, NJ 08102 USA; 2 Physics Department, Rutgers University, Camden, NJ 08102 USA; 3 Department of Mathematics and Computer Science, Southwest Minnesota State University, Marshall, MN 56258 USA; 4 Biology Department, Rutgers University, Camden, NJ 08102 USA

## Abstract

Evidence suggests that electroencephalographic (EEG) activity extends far beyond the traditional frequency range. Much of the prior study of >120 Hz EEG is in epileptic brains. In the current work, we measured EEG activity in the range of 200 to 2000 Hz, in the brains of healthy, spontaneously behaving rats. Both arrhythmic (1/f-type) and rhythmic (band) activities were identified and their properties shown to depend on EEG-defined stage of sleep/wakefulness. The inverse power law exponent of 1/f-type noise is shown to decrease from 3.08 in REM and 2.58 in NonREM to a value of 1.99 in the Waking state. Such a trend represents a transition from long- to short-term memory processes when examined in terms of the corresponding Hurst index. In addition, treating the 1/f-type activity as baseline noise reveals the presence of two, newly identified, high frequency EEG bands. The first band (ψ) is centered between 260–280 Hz; the second, and stronger, band is a broad peak in the 400–500 Hz range (termed ω). Both of these peaks display lognormal distributions. The functional significance of these frequency bands is supported by the variation in the strength of the peaks with EEG-defined sleep/wakefulness.

## Introduction

Global brain activity is conventionally measured in the electroencephalogram, which is comprised of oscillations in several functionally-relevant frequency bands. Historically, the bands were identified as δ (1–4 Hz), θ (4–7 Hz), α/μ (8–13 Hz), β (beta, 15–30 Hz), γ (gamma, 30–80 Hz) and high γ (80–150 Hz) waves^1,2^. In addition, “ripples” can be demonstrated as brief bouts of 80–200 Hz oscillations. “Fast ripples” are 250–600 Hz oscillations which occur in epileptogenic brain near the site of a lesion^2,3^. In sensory evoked potentials, oscillations are known to occur in the 200–400 Hz and higher ranges in rats^4^ and humans^5^. EEG in the range ≥200 Hz has been elicited by high-frequency stimulation of the thalamus in healthy rats^6^. However, non-pathological spontaneously-occurring EEG (i.e., not stimulus-evoked) over 200 Hz has not previously been reported^3^. Furthermore, the functions of high frequency EEG are not fully elucidated. The current study is inspired by a recently published theoretical model which proposed a role for high frequency brain activity as a critical factor for signal transmission in the brain^7^.

In addition to rhythmic EEG activity in the 200–1000 Hz range, 1/f-type noise is expected across the full range of brain activity examined. Here, the term “1/f-type” is used to indicate an inverse power law dependence (1/f^β^) that quite often displays behavior better described by an exponent (β) other than 1. The presence of 1/f-type noise in the conventional EEG spectral range (i.e. 1–100 Hz) has been noted several times over the past forty years^8–13^ and its presence in the higher frequency range would come as no surprise given its ubiquitous nature. The manifestation of 1/f-type behavior in the electrical activity of the brain has often been related in some part to ion channel activity fluctuations^14,15^. A recent study by Pettersen *et al*.^16^, suggests that power spectral densities (PSD) of such noise exhibit two different exponential dependencies and as such two different possible contributors. At lower frequencies, they argue that synaptic noise is the dominant contributor and at higher frequencies intrinsic channel noise dictates the value of the exponent. Other examples of such inverse power law dependency have been noted by Linkerkaer-Hansen *et al*.^17^, in the decay of μ and β amplitude fluctuations. It is important to remember that EEG is a macroscopic electrophysiological measurement that reflects summation of synchronized potentials within the cerebral cortex and therefore is insensitive to single channel fluctuations.

## Results

**Figure 1:**
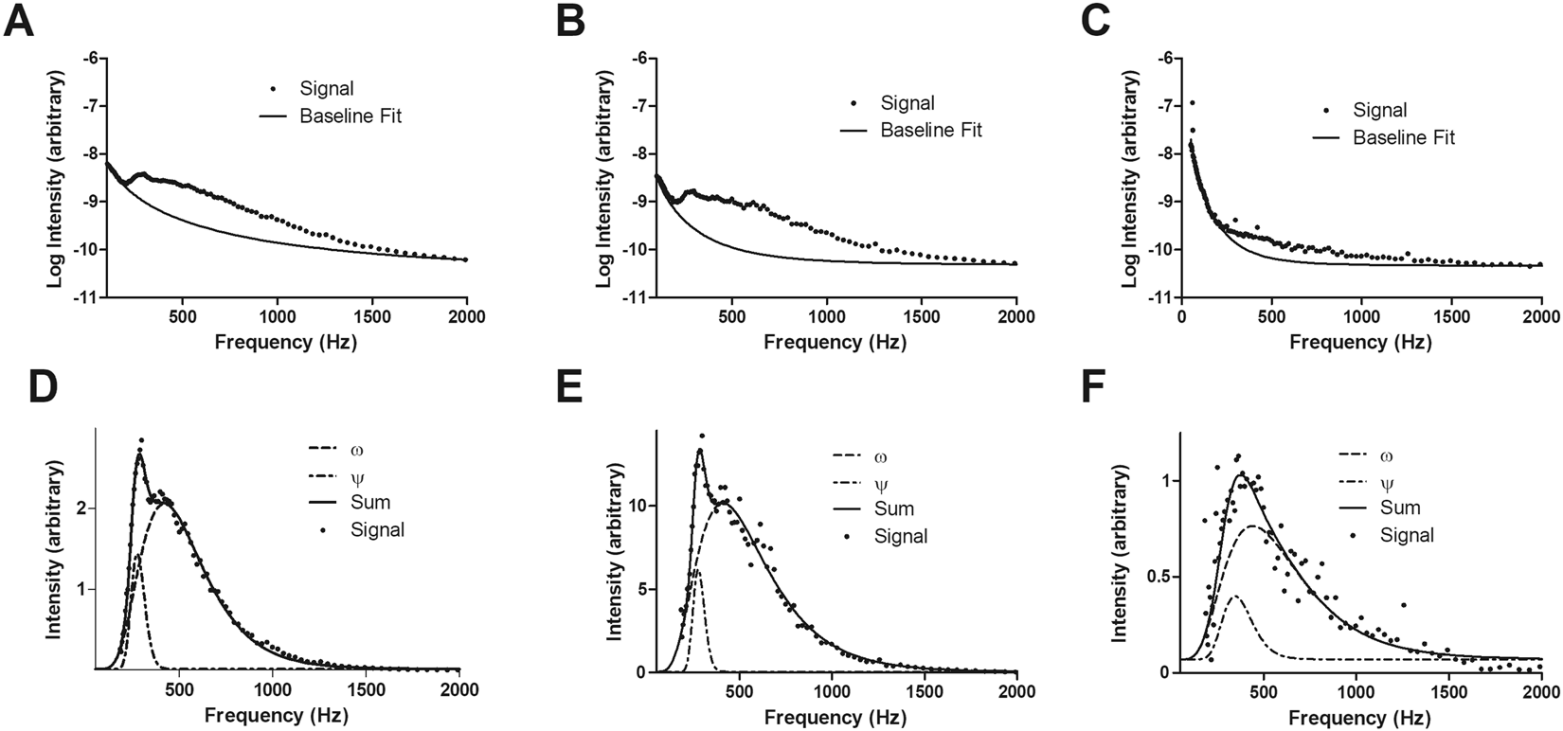
Summation of high-frequency brain activity signal data in different EEG states and characterization of ψ and ω frequency bands. Panels A–C show the overall high-frequency brain activity signal data (dots) from 100–2000 Hz as well as the 1/f-type baseline (solid). Each spectrum is an averaged Fourier analysis of high-frequency signal (1–2000 Hz) during EEG-defined intervals of waking (**A**), NonREM sleep (**B**), and REM sleep (**C**). Panels D–F show the signal (dots), along with curve-fitted sum (solid), as well as the peaks corresponding to the ψ band (lower trace) and the ω band (upper trace) during periods of waking (**D**), NonREM sleep (**E**), and REM sleep (**F**). The data are averages of results from 3 rats.

As shown in Fig. 1, brain EEG activity is apparent in the 200–1000 Hz PSD range. After correcting for 1/f-type noise (Fig. 1A–C), two regions of high-frequency EEG were visible (Fig. 1D–F) and display lognormal spectral distributions. The first distribution, which we have termed ψ, displayed a peak in the range of 285–315 Hz and the second distribution, termed ω, occurred in the 385–485 Hz range. The peaks varied with the EEG-defined stage of sleep/wakefulnesss, having much greater peak areas in Waking (Fig. 1D) than in NonREM (Fig. 1E) or REM (Fig. 1F) sleep. These will be referred as the *ψ*-band and *ω*-band, respectively. As noted in Table 1, the peak spectral location of the bands, during both waking and NonREM states, remain relatively unaltered; while, during REM their frequencies display increases by 4.9% and 21% with respect to waking. Upon waking from NonREM, both bands show a marked (~3-fold) increase in net strength.

**Table 1:** Frequency bands resulting from curve-fitting of high-frequency signal data in different EEG states.

EEG State	Peak Frequency (Hz^A^)	Median Frequency (Hz)	S.D.^B^ (Hz)	Strength (arbitrary^C^) × 10^−8^	H
Waking – ψ Band^D^	288	291	11.5	7.60	0.49
Waking – ω Band^E^	389	478	14.2	155	
NonREM – ψ Band	287	290	8.89	3.10	0.79
NonREM – ω Band	416	509	10.5	47.0	
REM – ψ Band	302	314	51.5	7.46	1.04
REM – ω Band	471	583	29.3	0.957	

^A^Values are determined as the mean data from three rats.

^B^S.D. is the standard deviation of the median frequency. ^C^The strength is represented by the area under curve. ^D^The ψ band is defined by the frequency range 200–350 Hz. ^E^The ω band is represented by the frequency range 375–1000 Hz.

A one-way ANOVA showed that the strength of the ω band was significantly influenced by the EEG state (F = 16.18; P = 0.0121) and Bonferroni’s *post hoc* tests indicated that the strength of the band was significantly different at the P < 0.05 level between Wake and NonREM and between Wake and REM. The ANOVA for the effect of EEG state on the strength of the ψ band showed a trend toward significance (F = 4.58; P = 0.092). The one-way ANOVA of the median peak frequency of the ω band showed a significant effect of EEG-defined state (F = 16.04; P = 0.0123) and post-hoc Bonferroni’s multiple comparison tests indicated a significant shift in frequency between Wake and REM states (P < 0.05). The median peak frequency of the ψ band was not significantly influenced by EEG-defined state (F = 0.4768; P = 0.652).

**Figure 2:**
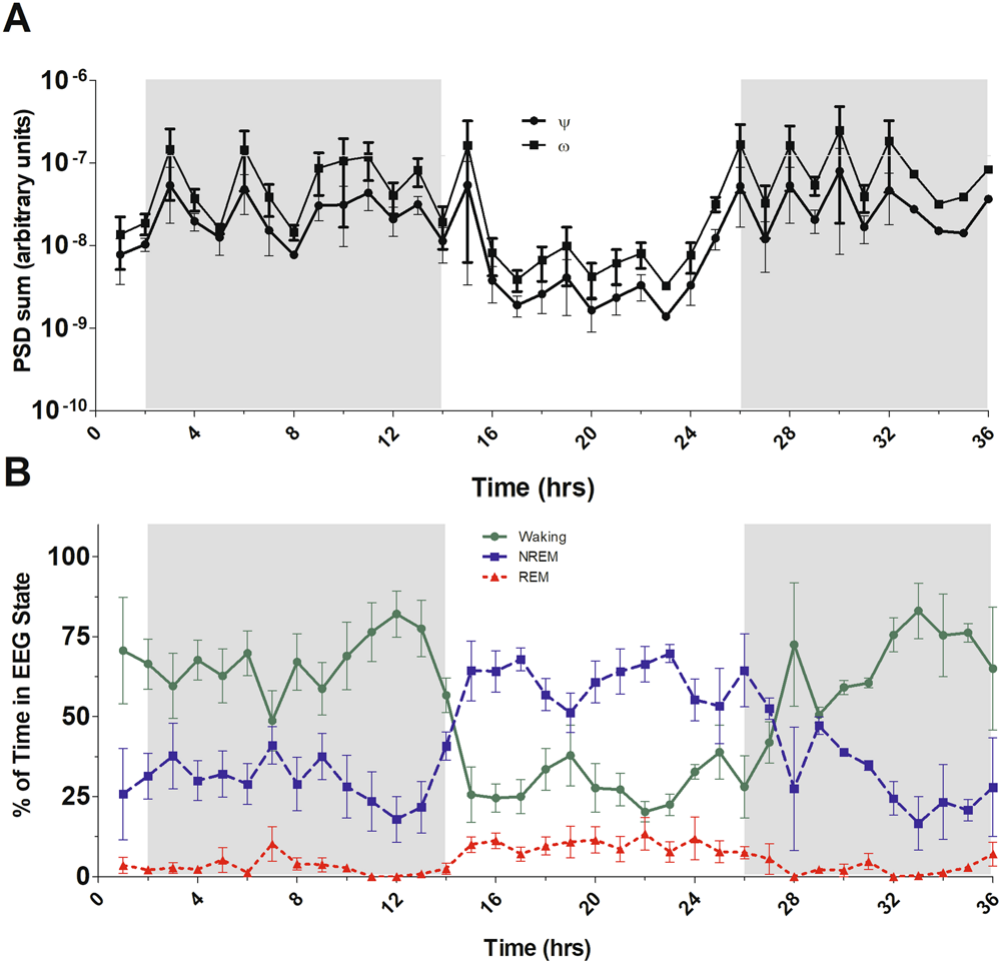
Power spectrum density of ψ and ω and percentage of mean wakefulness, NonREM sleep, and REM sleep for each one-hour period over 36 hours. The power spectrum density sum was calculated for each hour over 36 hours. Panel (A) represents the ψ frequency band (285–315 Hz, squares) and the ω frequency band (385–585 Hz, filled circles). Panel (B) shows the percentage of mean wakefulness (solid with filled circles), NonREM sleep (dashes with squares), and REM sleep (dots with triangles) over 36 hours. The shaded area denotes the 12-hour dark phase of the light-dark cycle. The data are average results from three rats and the error bars indicate standard errors of the mean.

EEG-defined Waking is higher in the dark phase of the light-dark cycle (Fig. 2; lower panel) while REM and NonREM sleep are higher in the light phase (Fig. 2, lower panel) of this nocturnal animal. The power spectrum density of both ψ and ω were lower in the light phase of the light-dark cycle (Fig. 2; upper panel).

## Discussion

As reviewed by Buzsáki and Mizuseki^18^, the presence of skewed distributions with heavy-tails, such as a lognormal, are quite common in synaptic firing rates and brain activity in general since such systems are often multiplicative in nature. Through the use of high-frequency data collection in conjunction with conventional EEG collection in adult male rats, we demonstrate the existence of novel, bimodal brain oscillations centered at the ~280 Hz (ψ) and 400–500 Hz (ω) frequency ranges. These oscillations are present after the removal of 1/f-type noise, and show differing characteristics in different EEG-defined states. The general PSD sum also showed a consistently higher average value during the active period of the rats. The values of the PSD sum in *ψ* and *ω* bands increased during periods of wakefulness. The PSD sum changes along with the shifts in the most prominent state of EEG (Wakefulness, REM or NonREM) over time, indicating a diurnal cycle, presumably tied to the light-dark cycle.

The *1/f-type* baseline exponent decreases from 3.08 − 2.58 − 1.99 for REM, NonREM, and Waking respectively. Large values for exponents approaching 3 are consistent with the literature for EEG^9^. In terms of fractal dimensional analysis, it is common to describe the exponent in terms of the Hurst index (H) which is defined as H = |β − 1|/2. The H parameter is used as a means of gauging the degree of dependence of stochastically-related events. A Hurst index of H ∈ (0.5,1) represents long-range stochastically dependent increments, a H = 0.5 has stochastically independent increments and a H ∈ (0,0.5) signifies short-range stochastically dependent increments (see^19,20^). While the influence of waking state on the Hurst index of high frequency EEG activity is not the premise of the current study it is nevertheless interesting to note that the H seems to decrease with increasing wakefulness implying that the underlying processes become less reliant on past events. A lower Hurst index shown during Waking (0.49) than during NonREM sleep (0.79) or REM sleep (1.04) may indicate a difference in the stochastic interdependence during those discrete EEG-defined states. The Hurst index for Waking indicates a short-range stochastically dependent increment (nearly stochastically independent), while the REM and NonREM Hurst indices indicate long-range stochastically dependent increments.

Our finding of new bands of high-frequency activity in the EEG of healthy, spontaneously behaving rats provides additional details in the picture of the types of possible brain oscillations. The two bands, termed ψ and ω, vary in strength with the state of EEG (Wakefulness, REM Sleep or NonREM Sleep), suggesting new correlates of wakefulness that might have practical utility in clinical studies. The presence of *1/f-type* noise in the high-frequency recording echoes the findings of *1/f-type* noise in the lower frequency EEG, emphasizing the ubiquity of this phenomenon in brain activity. The finding of new frequency bands will open new areas of investigation of the functionality of high frequency EEG.

## Methods

### Measurement of EEG-defined sleep and waking

All animal use was approved by the Rutgers University Institutional Animal Care and Use Committee. The care and use of the animals was according to the stipulations of this committee. Adult male Sprague-Dawley rats (Hilltop Lab Animals, Scottdale, PA) were housed individually, given food and water *ad libitum* and handled frequently to reduce the effects of stress. The temperature of the facility was maintained at 22.2–23.3 °C on a 12 hour light/12 hour dark cycle (lights on at 07:00). Rats were anesthetized with isoflurane using an EZ Anesthesia vaporizer apparatus. Next, an incision was made in the scalp, the skull exposed, and a screw EEG electrode (Plastics One E363-20) inserted in each quadrant of the skull. Two Teflon-coated wires with stripped ends were inserted into the neck musculature to serve as EMG electrodes. The electrode sockets were inserted into an electrode pedestal (Plastics One MS363), then secured with dental acrylic.

After a recovery period of a week, animals were placed in an individual chamber within a shielded room and connected to a multichannel amplifier (Grass Instruments Model 15) via a shielded cable leading through a multichannel commutator (Plastics One SL6C). At this point, one of the channels was connected to the Hewlett-Packard model 3562 A signal analyzer for recording of brain activity of up to 1 kHz. In parallel, EEG and EMG were digitized using a data acquisition unit (CED Micro 1401) and recorded for 48 hours using Spike2 Software.

EEG and EMG data were analyzed offline by a trained researcher. Each record was evaluated in 30-second epochs, and a state of arousal (NonREM, REM, or Waking) assigned according to standard criteria^21^. High-frequency brain activity was recorded as described below.

### Measurement of high-frequency brain activity

The use of cranial implanted electrodes improves signal strength and reduces low-pass filtering effects that would otherwise hinder detection of high frequency neural oscillations. A single channel from the commutator was sent to a coaxial BNC breakout box, built to accept the 6-pin connector terminating the Plastics One electrode cable bundle, thereby facilitating individual electrode pair selection. In this study, measurements were conducted between the left frontal and left occipital electrodes.

Neural activity sensed by the electrodes was sent from the breakout box to a custom-built preamplifier stage. The preamplifier was built around a Linear Technologies LTC1051 zero-drift operational amplifier. The LTC1051 displays excellent DC and AC characteristics over the frequency range of interest (1 Hz–10 kHz) and utilizes chopper-stabilization in conjunction with internal capacitors to achieve low output noise ($1.5\,\mu {V}_{P-P}$). To further curtail instrumentation noise, the circuitry was powered by two 9 V batteries which fed a Texas Instruments TLE2426 rail splitter. The function of the rail splitter is to produce precise virtual ground positioned at one-half that of the single-supply battery source. Metal film resistors were used to minimize circuit 1/f noise; while, EMF noise was suppressed by housing the circuitry housed in a grounded aluminum cast case. Input and output connections were made using floating-shield coaxial BNC feedthroughs. The preamplifier was set to a nominal gain of 100x in order to circumvent saturation from lower frequency brain electrical activity (e.g., α rhythms) which can be a couple of orders of magnitude larger than the high-frequency brain activity. After amplification, the signal was ultimately sampled by a fast Fourier transform (FFT) based dynamic signal analyzer (Hewlett-Packard model 3562 A). A frequency range starting at 50 Hz and spanning two decades was chosen along with log resolution to minimize acquisition time and optimize data collection in the region of interest.

### 1/f noise subtraction and log-normal fits

In order to decouple high frequency EEG activity from the *1/f-type* noise, in this case acting as a baseline, the low (<100 Hz) and high (>1000 Hz) regions of the PSD spectra were isolated and fit using regression analysis to a power law, see Fig. 1. This defines a baseline that was subtracted from the PSD spectra in order to resolve the presence of RRF activity. As also seen in Fig. 1, the subsequent spectra are bimodal in nature and display a log-normal distribution. Each mode (band) was deconvolved via spectral fitting to a log-normal distribution to determine their relative strengthens, bandwidths, and dependences on waking state, Fig. 1. As reviewed by Buzsaki, and Mizuseki [12], the presence of skewed distributions with heavy-tails, such as a lognormal, are quite common in synaptic firing rates and neurological activity in general since such systems are often multiplicative in nature. Due to the lower occurrence of REM events over the collection period than wake and/or NonREM states, the data set for high-frequency brain activity activity during REM displays increased statistical noise.

### Data availability

The datasets generated and analyzed during the current study are available from the corresponding author on reasonable request.

## References

[1] Groppe DM (2013). Dominant frequencies of resting human brain activity as measured by the electrocorticogram. NeuroImage.

[2] Buzsáki, G. *Rhythms of the Brain*. (Oxford University Press, 2006).

[3] Engel J, Bragin A, Staba R, Mody I (2009). High-frequency oscillations: What is normal and what is not?. Epilepsia.

[4] Jones MS, MacDonald KD, Choi B, Dudek FE, Barth DS (2000). Intracellular correlates of fast (>200 Hz) electrical oscillations in rat somatosensory cortex. Journal of Neurophysiology.

[5] Klostermann, F., Gobbele, R., Buchner, H. & Curio, G. Intrathalamic non-propagating generators of high-frequency (1000 Hz) somatosensory evoked potential (SEP) bursts recorded subcortically in man. *Clinical Neurophysiolog*y **11**3, 1001-1005, Pii S1388-2457(02)00119-0. 10.1016/S1388-2457(02)00119-0 (2002). 10.1016/s1388-2457(02)00119-0 12088692

[6] Kandel A, Buzsaki G (1997). Cellular-synaptic generation of sleep spindles, spike-and-wave discharges, and evoked thalamocortical responses in the neocortex of the rat. J. Neurosci..

[7] Hong D, Man S, Martin JV (2016). A stochastic mechanism for signal propagation in the brain: Force of rapid random fluctuations in membrane potentials of individual neurons. J Theor Biol.

[8] Grigolini P, Aquino G, Bologna M, Lukovic M, West BJ (2009). A theory of 1/f noise in human cognition. Physica A.

[9] He BYJ (2014). Scale-free brain activity: past, present, and future. Trends in Cognitive Sciences.

[10] Kamitani, Y. & Matsuba, I. Neural networks in three dimensions producing 1/f spectra. *8th International Conference on Neural Information Processing, Vols 1-3, Proceeding*, 627–631 (2001).

[11] Pereda E, Gamundi A, Rial R, Gonzalez J (1998). Non-linear behaviour of human EEG: fractal exponent versus correlation dimension in awake and sleep stages. Neurosci Lett.

[12] Pritchard WS (1992). The brain in fractal time - 1/F-like power spectrum scaling of the human electroencephalogram. Int J Neurosci.

[13] Voytek B (2015). Age-Related Changes in 1/f Neural Electrophysiological Noise. J. Neurosci..

[14] Clay JR, Shlesinger MF (1976). Theoretical model of the ionic mechanism of 1/f noise in nerve membrane. Biophys J.

[15] Siwy, Z. & Fulinski, A. Origin of 1/f(alpha) noise in membrane channel currents. *Physical Review Letter*s **89**, 10.1103/PhysRevLett.89.158101 (2002). 10.1103/PhysRevLett.89.158101 12366027

[16] Pettersen KH, Linden H, Tetzlaff T, Einevoll GT (2014). Power laws from linear neuronal cable theory: power spectral densities of the soma potential, soma membrane current and single-neuron contribution to the EEG. Plos Comput Biol.

[17] Linkenkaer-Hansen K, Nikouline VV, Palva JM, Ilmoniemi RJ (2001). Long-range temporal correlations and scaling behavior in human brain oscillations. J Neurosci.

[18] Buzsaki G, Mizuseki K (2014). The log-dynamic brain: how skewed distributions affect network operations. Nat Rev Neurosci.

[19] Mandelbrot BB, Van Ness JW (1968). Fractional Brownian motions, fractional noises and applications. SIAM Review.

[20] Mishura, Y. *Stochastic Calculus for Fractional Brownian Motion and Related Processes*. 1–393 (Springer, 2007).

[21] Martin JV, Cook JM, Hagen TJ, Mendelson WB (1989). Inhibition of sleep and benzodiazepine receptor binding by a beta-carboline derivative. Pharmacology Biochemistry and Behavior.

